# Fluorinated reduced graphene oxide as a protective layer on the metallic lithium for application in the high energy batteries

**DOI:** 10.1038/s41598-018-23991-2

**Published:** 2018-04-11

**Authors:** Jernej Bobnar, Matic Lozinšek, Gregor Kapun, Christian Njel, Rémi Dedryvère, Boštjan Genorio, Robert Dominko

**Affiliations:** 1 National Institute of Chemistry, Hajdrihova 19, SI-1001 Ljubljana, Slovenia; 2 University of Ljubljana, Faculty of Chemistry and Chemical Technology, Večna pot 113, SI-1001 Ljubljana, Slovenia; 3 Jožef Stefan Institute, Jamova cesta 39, SI-1000 Ljubljana, Slovenia; 4 IPREM-ECP (UMR 5254 CNRS), University of Pau, Hélioparc, 2 av. Pierre Angot, 64053 Pau Cedex 9, France; 5 ALISTORE - European Research Institute, 33 rue Saint-Leu, 80039 Amiens Cedex, France

## Abstract

Metallic lithium is considered to be one of the most promising anode materials since it offers high volumetric and gravimetric energy densities when combined with high-voltage or high-capacity cathodes. However, the main impediment to the practical applications of metallic lithium is its unstable solid electrolyte interface (SEI), which results in constant lithium consumption for the formation of fresh SEI, together with lithium dendritic growth during electrochemical cycling. Here we present the electrochemical performance of a fluorinated reduced graphene oxide interlayer (FGI) on the metallic lithium surface, tested in lithium symmetrical cells and in combination with two different cathode materials. The FGI on the metallic lithium exhibit two roles, firstly it acts as a Li-ion conductive layer and electronic insulator and secondly, it effectively suppresses the formation of high surface area lithium (HSAL). An enhanced electrochemical performance of the full cell battery system with two different types of cathodes was shown in the carbonate or in the ether based electrolytes. The presented results indicate a potential application in future secondary Li-metal batteries.

## Introduction

Current Li-ion technology relies on intercalation or conversion anodes with limited theoretical energy densities. In order to launch batteries with an even higher energy density we need to introduce lithium metal as a negative electrode. Lithium metal can, in theory, significantly improve the energy density of cells when combined with classical insertion or conversion cathode materials as well; it is the best choice if we want to employ post lithium ion battery chemistry^1,2^. However, secondary lithium-metal batteries still suffer from the low Coulombic efficiency and low safety related to the thermodynamically unstable interface between the metallic lithium and electrolyte in majority of the liquid electrolytes.

The interaction between the lithium metal and electrolyte leads to the formation of mechanically unstable heterogeneous solid electrolyte interface (SEI) on the lithium-metal surface. It was shown that during stripping and deposition, SEI mechanically breaks and cracks. In these cracks, the fresh lithium surface is exposed to the electrolyte and a new thinner SEI is formed. The surface of the thin SEI layer is the preferred area for successive deposition of lithium during battery cycling. It is widely accepted that the main reason for selective deposition is a higher electrical field on the cracked areas of the metal anode^3,4^. This cyclic process usually manifests in the formation of dendrites and mossy lithium, which are defined as a high surface area lithium (HSAL)^5,6^.

Formation of HSAL and continuous consumption of the electrolyte due to fresh surface formation are the main causes for lithium-metal battery failure. This can be suppressed by different approaches e.g. a) mechanically increasing the lithium surface area (lithium metal hybrid anode^7–9^, lithium alloying^10–12^, and stabilized lithium powder^13^), b) by using solid electrolytes with a high Young’s modulus^14^, c) introducing additives in the electrolyte, which react primarily with the lithium metal, enabling formation of the *in situ* SEI which prevents further degradation of the electrolyte on the lithium surface (e.g. LiNO_3_, CO_2_, and SO_2_)^15^ and d) pre-treating the lithium-metal surface with various materials in order to prepare *ex situ* formatted artificial SEI (porous carbons^16,17^, polymers^18–21^, ceramic layers^22^, and inorganic-organic composites^23–25^).

*Ex-situ* formation of an artificial SEI is used to prepare an engineered SEI which must be: a) as thin as possible, b) highly ionically conductive, c) electronically non-conductive, and d) a material with high Young’s (elastic) modulus in order to withstand the stress applied during battery cycling. All the proposed properties are important for long-term operation of the battery.

Graphene and its derivatives are promising materials which could meet the expectations for artificial SEI applications due to their high Young’s modulus^26^. Additionally, by proper functionalization of graphene, other properties can be tuned or modified^27^. Electronic properties can be altered by the functionalization of graphene basal planes or introduction of the structural defects. Similarly, the lithium-ion permeability is also widely affected by the introduction of structural defects on the graphene layer^28,29^. The study of the diffusion mechanism of lithium ions has shown that it is perpendicular to the basal plane of graphene and it is facilitated by the increased number of defects, while the diffusion parallel to the plane is limited by the steric hindrance that originates from aggregated lithium ions adsorbed on the abundant defect sites^30^. Due to their unique properties, graphene derivatives have already been tested in battery applications in reference to dendritic-growth suppression. The majority of studies have focused on graphene deposition layers on the separator^31,32^ or on the current collector (Cu foil)^33,34^, where it was shown that induced topological defects enable mobility of the lithium ion through these structural defects and consequently suppresses dendritic growth.

A technologically interesting approach for lithium dendritic growth suppression is the direct protection of lithium metal. Zhang *et al*.^35^ tested reduced graphene oxide (rGO) as a dendrite suppression layer. They prepared thin graphene oxide (GO) film, which was reduced and pressed together with lithium metal. An interconnected rGO/Li composite electrode exhibited improved cycling stability in a Li-symmetrical cell compared to non-protected lithium. Furthermore, interconnected rGO film was able to suppress dendritic growth and also to store detached lithium. In the follow-up study, they used a drop-coating technique for the preparation of an artificial GO SEI and they observed enhanced electrochemical performance in a Li-S battery and better cycling stability in Li symmetrical cells compared to a non-protected Li-metal electrode^36^. Additionally, by using GO as an artificial SEI, the side reactions between the electrolyte and lithium anode were inhibited, however, a mossy-like morphology on the lithium surface was still observed. All previously described attempts on engineered artificial SEIs to suppress dendritic growth were just partially effective^4,15,37^. In most of the described studies, HSAL was still observed. HSAL represents a hazard for short circuits in the battery and additionally, it is the main cause of constant electrolyte consumption, which leads to the increased overpotential and low Coulombic efficiency due to battery “dry out”. High-energy density batteries operate with a low amount of electrolyte; hence, the degradation of electrolyte leads to premature failure of the battery. Therefore, an efficient artificial SEI should completely prevent HSAL formation, including the formation of dendrites which should enable the long-term cyclability of batteries with low electrolyte content. In this respect, efficient lithium-anode protection still remains an unsolved issue and a topic of research.

Herein we report on the impact of fluorinated reduced graphene oxide (FG) drop casted on the lithium surface as an interlayer FG prevents formation of HSAL during electrochemical stripping and deposition. Fluorinated graphene exhibits a high Young’s modulus (0.3 TPa) and a resistance exceeding 10 GΩ at room temperature^27^. Furthermore, fluoro-functionalized rGO coated on a separator has been shown to have a positive effect on the capacity stabilization in Li-S batteries^38,39^. Aforementioned properties motivated us to prepare, characterize, and test FG material with an increased number of structural defects as an interlayer between the electrolyte and metallic lithium. Properties of the FGI (fluorinated reduced graphene oxide interlayer) have been characterized by electron microscopy (SEM and FIB-SEM), XPS, and electrochemically tested in the symmetrical cell and in combination with two different cathode materials.

## Results and Discussion

### Fluorinated reduced graphene oxide interlayer on Li-metal (FGI@Li)

**Figure 1:**
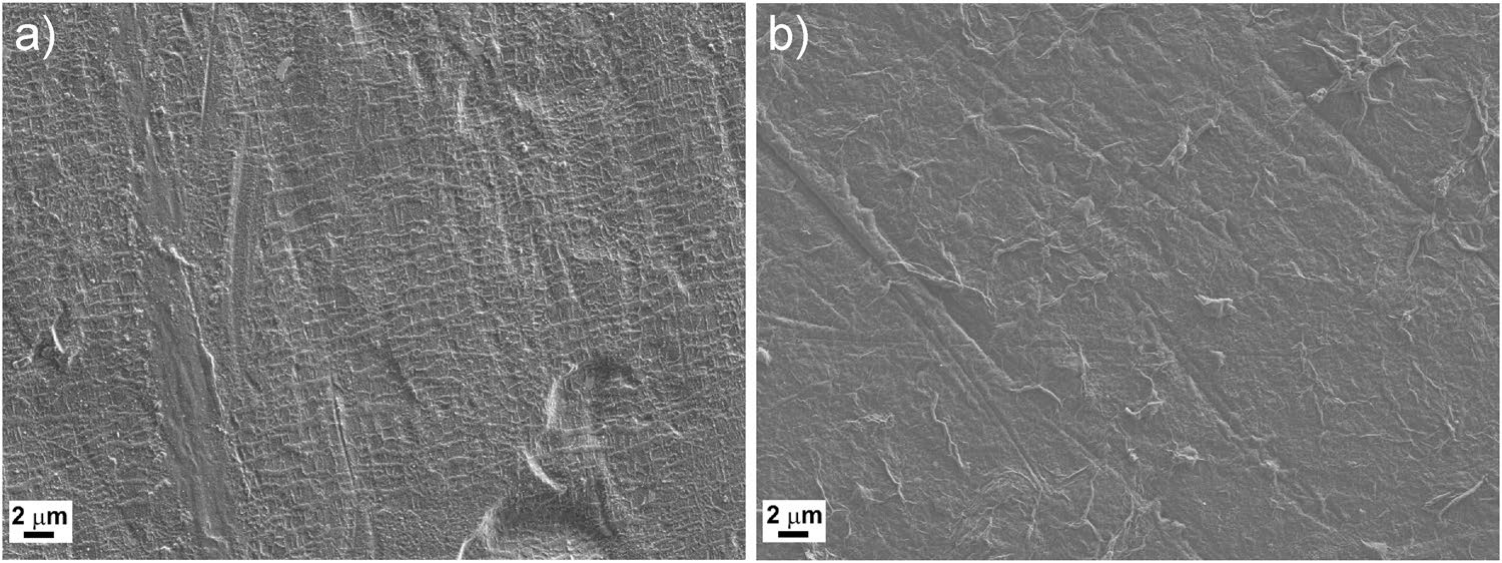
Morphology comparison of non-protected and FGI protected lithium. SEM images of (**a**) non-protected lithium surface and (**b**) FGI@Li.

The solvent for preparation of FG dispersion was selected by optimizing key parameters. Selection conditions were as follows: a) maximum stability towards lithium metal and b) good dispersability and exfoliability for the FG flakes. Propylene carbonate (PC) proved itself to be the best tradeoff between required properties. The organic solvent PC is not aggressively reactive to lithium metal and it is able to disperse and exfoliate FG flakes in concentrations up to 0.5 mg mL^−1^ (Supplementary Figure S1). The preparation of an artificial FGI was achieved by drop casting the FG dispersion (from 0.19 up to 0.21 mg mL^−1^) on a Li-foil surface and removing of the solvent under a protective atmosphere in vacuo. The change of surface morphology between the non-protected lithium and the FGI@Li was examined by SEM and it is shown in Fig. 1. The non-protected lithium (Fig. 1a) exhibits ripple marks on the surface (similar to ripples in a desert sand bedform). These ripples seem to be masked by an artificial FGI; however, wrinkle-like features are visible on FGI@Li (Fig. 1b). Based on mass loading (10 μg cm^−2^), the obtained coating is multilayered with stacked FG flakes that can be observed as wrinkles, which are typical features when several layers of graphene sheets are distributed on flat substrates^40,41^.

The energy-dispersive X-ray spectroscopy (EDX) analysis was done on FG flakes, pristine lithium, and FGI@Li (Supplementary Figure S2). EDX analyses show that FG is composed of carbon, oxygen, and fluorine with an atomic ratio of 1.8:0.2:1.0. Carbon, oxygen, and sulfur were detected on the pristine lithium surface. In contrast, after lithium is protected by FGI, EDX detected carbon, oxygen, and fluorine with an atomic ratio of 3.5:2.6:1.0. An additional signal for carbon and oxygen was assigned to the passive layer of the lithium foil used in our experiments.

### Lithium metal symmetrical cell

Two different types of the symmetrical cells were assembled, namely non-protected lithium and FGI@Li, both with an excess of the electrolyte (80 μL per cell, corresponding to the 27 μL cm^−2^). Cells were electrochemically evaluated to see the impact of the FGI on the stability during stripping and deposition (increase of polarization and formation of short circuits).

**Figure 2:**
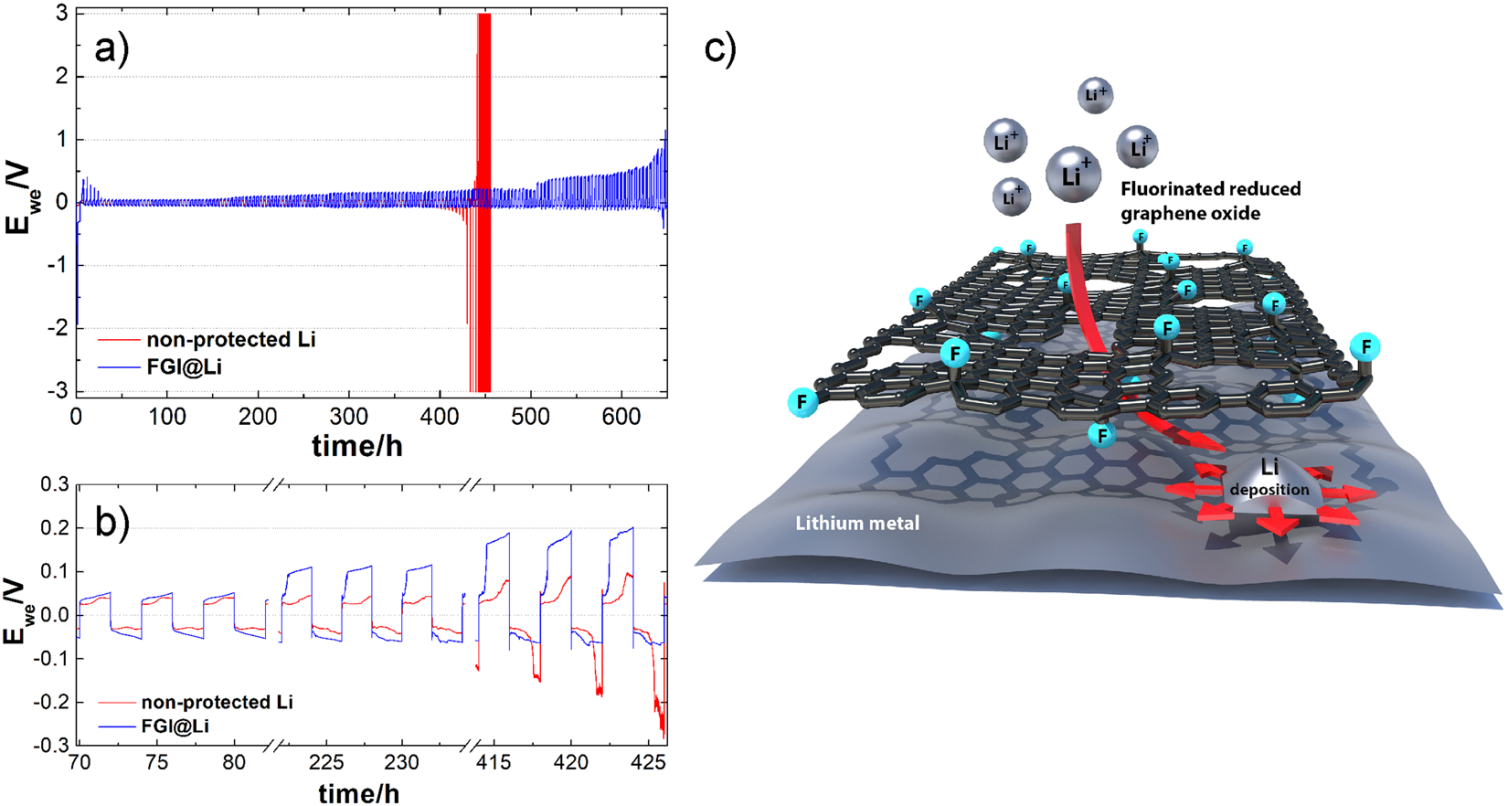
Voltage versus time curves for Li-symmetrical cells and shematics of lithium deposition on FGI@Li. (**a**) A comparison of the electrochemical stability of the FGI@Li (blue) and non-protected Li (red) with excess of the ether based electrolyte (27 μL cm^−^^2^) at a current density of 0.5 mA cm^−2^. (**b**) Enlarged ranges from 70 h to 82 h, 222 h to 234 h, and 414 h to 426 h. (**c**) Schematic illustration of Li-ion penetration through the FGI and lithium deposition onto lithium surface underneath the FGI.

Figure 2 shows a comparison of the electrochemical performance of non-protected lithium and FGI@Li. The Li-symmetrical cell with non-protected lithium shows stable cycling behavior up to 450 h, when polarization during continuous stripping and deposition rapidly increases due to the complete disintegration of the lithium electrode used in the measurement. The electrochemical behavior of non-protected lithium observed in our experiment is in agreement with results published by other groups^42–44^. The starting part of the deposition process is due to the nucleation process and after the polarization is decreased, the process of nucleation is changed by dendritic growth. The second peak corresponds to the process where stripping from HSAL is changed to stripping from the bulk. The symmetrical cell with FGI@Li electrodes shows increased polarization during the first five cycles (Supplementary Figure S3), which we ascribe to the formation of transport paths through FGI (Fig. 2a). Once all conduction paths are established, the overpotential drops close to the one observed with non-protected lithium with small additional polarization attributed to the stripping of lithium from underneath the FGI. With continuous cycling, the overpotential within the symmetrical cell with FGI@Li electrodes monotonously increases. Interestingly, the increase is faster in one direction compared to the other, where a more stable overpotential for the whole period of cycling can be observed (Fig. 2b). We correlate the observed increase of the overpotential with the formation of a passive layer on the FGI and potentially with the formation of HSAL in the imperfections of the interlayer. In comparison to non-protected lithium, an electrochemical curve for FGI@Li has a different shape. The curve is flatter and the overpotential is increasing gradually to the end half of the cycle. We propose that the FGI@Li electrode has a broader nucleation and stripping peak, which is due to the stripping of lithium from the underneath of FGI. These two broader peaks are merged together which is manifested in slowly increasing the polarization. According to literature^30,33,34^ and results discussed above, we hypothesize, that Li-ions penetrate through the structural defects of FGI and deposit directly onto the lithium surface underneath the FGI, as depicted on the Fig. 2c. Overall, the results in Fig. 2 demonstrate that the lifetime of the FGI@Li in the symmetrical cell is considerably longer which is due to the protective nature of the FGI. Here we can conclude that FGI serves as a protective layer between metallic lithium and the liquid electrolyte. This can be explained by the high Young’s modulus and high electronic resistance of fluorinated graphene. Additionally, fluorinated graphene is chemically resistant since the C–F bond is one of the strongest single bonds in organic chemistry. In contrast, the use of GO as an interlayer for protection of lithium shows that GO is easily reduced to rGO in the voltage window applied in the experiment, since stripping and deposition cycling was stopped after 46 cycles due to the formation of dendrites formed on the surface of electronically conductive rGO (Supplementary Figure S4).

**Figure 3:**
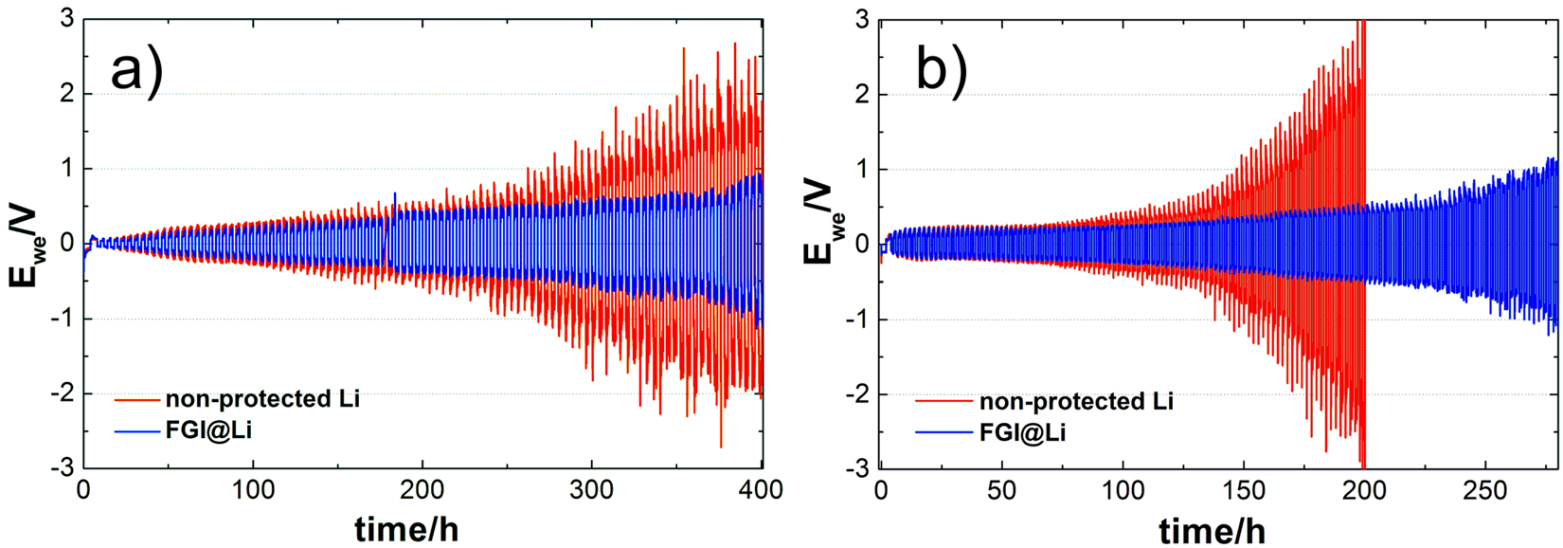
Voltage versus time curves for Li-symmetrical cells with reduced amount of electrolyte. A comparison of the electrochemical stability of the FGI@Li (blue) and non-protected Li (red) with a reduced amount of the electrolyte (3.3 μL cm^−2^) at a current density of (**a**) 0.5 mA cm^−2^ and (**b**) 1.0 mA cm^−2^.

Results with an excess of the electrolyte show significant improvement in terms of cycle life by using FGI@Li compared to the non-protected lithium. Previous reports on lithium-protection techniques paid no attention to the electrolyte quantity: either no information about the electrolyte volume quantity was reported^35,36,45^ or an excess of electrolyte was used^12,17,25^. The quantities of electrolyte varied from 30 µL to 50 µL with an electrode surface from 1 to 2 cm^2^. The practical amount of electrolyte should be less than 5 µL cm^−2^, in order to obtain high energy density cells. To simulate realistic conditions within the electrochemical cell, we reduced the amount of electrolyte and consequently we exchanged one fibrous separator with a thickness of 150 μm with a layer of Celgard (that reduced the available amount of electrolyte by a factor of 10). The volume of the electrolyte was reduced to 10 μL per cell corresponding to 3.3 μL cm^−2^ (normalized to the available lithium surface at the beginning of experiment). This enabled the simulation of more realistic conditions where a problem due to increased polarization connected to electrolyte consumption can be expected. Comparison of the stripping and deposition tests between non-protected lithium and FGI@Li performed with a reduced amount of electrolyte are depicted in Fig. 3a. Results revealed that overpotential of symmetrical cell with non-protected lithium increases rapidly and reaches 2 V after 400 h of the stripping and deposition test. In contrast, the symmetrical cell with FGI@Li shows a more stable overpotential which increases slowly. After 400 h of cycling, the overpotential of the FGI@Li cell is only half of that observed with non-protected lithium. The standard deviation calculated from set of measurements reveals even with quite high error for both non-protected Li and FGI@Li, that FGI@Li has lower overpotential (0.8 V ± 0.3 V) compared to non-protected Li (1.5 V ± 0.2 V) after 400 hours. Furthermore, FGI@Li was compared to non-protected Li in Li-symmetrical cell with the same configuration at higher current density of 1 mA cm^−2^ (Fig. 3b). At higher current density overpotential increase even more rapidly for symmetrical cell with non-protected lithium and reach cut-off voltage at 3 V in 200 h. Symmetrical cell with FGI@Li shows more stable cycling behavior and reaches significantly lower overpotential (0.6 V) after 200 h of lithium stripping/deposition test. Thus, a faster increase of the polarization of the non-protected lithium cell is due to electrolyte decomposition on the surface of the fresh HSAL. It is well accepted^17^ that consumption of the electrolyte during stripping and deposition is reduced when lithium is coated by a more stable SEI. Consequently, HSAL growing on FGI@Li is considerably less pronounced and the system is more robust. To show the range of applicability of FGI@Li, its electrochemical behavior was further evaluated in the same configuration in Li symmetrical cell with LP40 electrolyte (Supplementary Figure S5). FGI@Li exhibits more stable and longer cyclability also in carbonate-based electrolyte compared to the non-protected Li. Symmetrical cells with non-protected lithium have stable stripping and deposition for less than 100 hours and afterwards spikes can be observed in the signal. In contrast, FGI@Li shows stable cycling behavior with lower overpotential at 100 hours. Stable cycling behavior of FGI@Li continues to 170 hours when spikes can be observed.

FGI is stable in the voltage window applied in the experiment and it represents a protective passive film that suppresses the formation of HSAL due to the insulating properties of the FG. Furthermore, fluorine impurities (free-trapped fluorine species)^39^ can react and form the LiF as a side product on the surface of metallic lithium, which is also favorable due to good ionic conductivity and high electrical resistance of LiF^46^. The aforementioned result was confirmed by electrochemical stripping/deposition tests, where FG protected lithium shows an enhanced stability and slower increase of polarization during cycling even with a reduced amount of electrolyte.

### Post-mortem analyses

**Figure 4:**
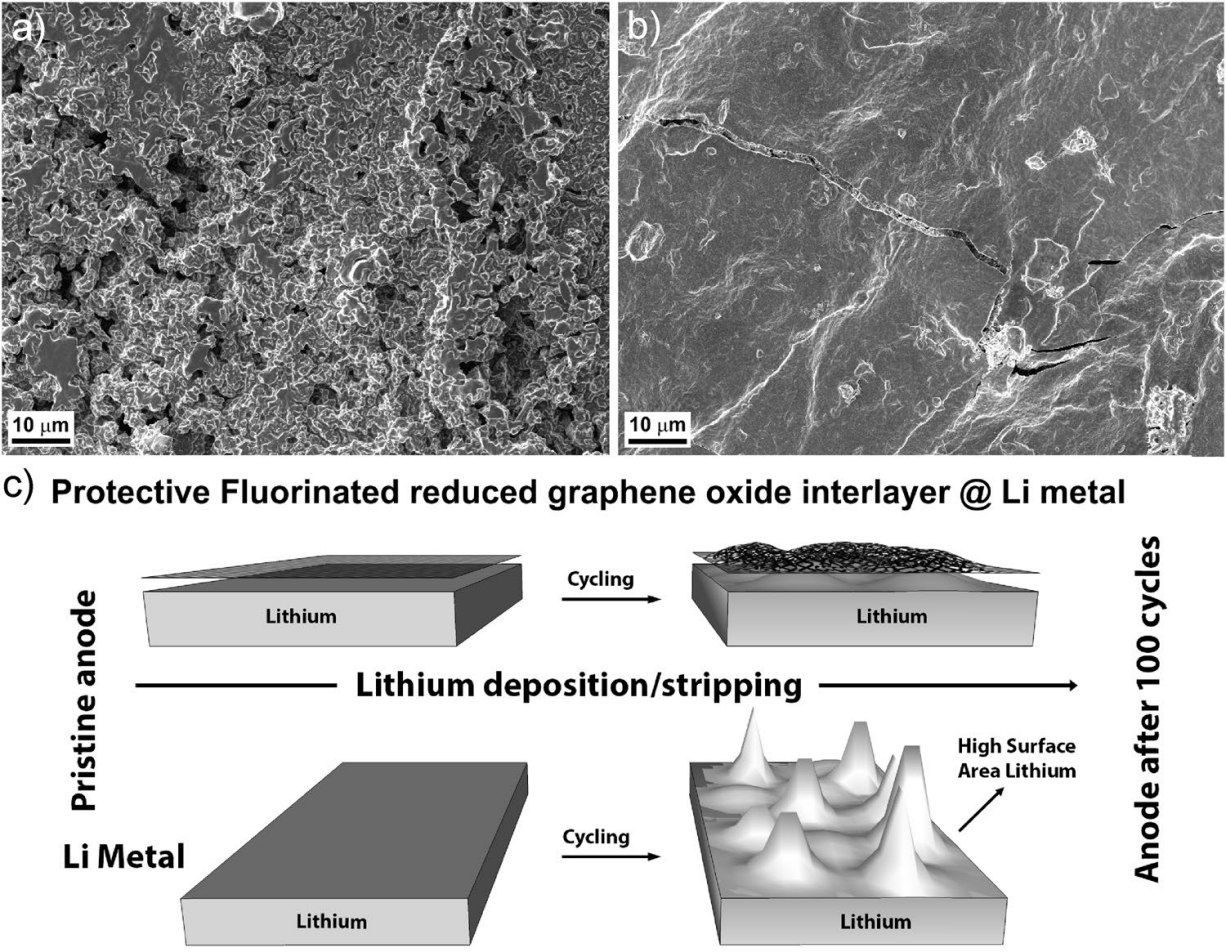
Post-mortem morphology and schematic representation. SEM images of the lithium electrodes after 100 cycles (**a**) non-protected lithium foil, (**b**) FGI@Li, and (**c**) schematic representation of the morphological changes on non-protected lithium and FGI@Li electrodes.

The morphological changes of the non-protected lithium and FGI@Li after 100 cycles of stripping and deposition were analyzed by SEM and FIB-SEM. Figure 4a shows the morphology of the non-protected lithium surface after cycling which is significantly changed compared to a fresh lithium surface before cycling (Fig. 1a). HSAL is clearly visible on the surface of Li-metal (similar morphology was already observed after 30 cycles as shown in Supplementary Figure S6). On the other hand, the morphology of the FGI@Li electrode surface after 100 cycles shown in Fig. 4b reveals only minor differences compared to Fig. 1b. The observed morphological differences are schematically represented in Fig. 4c. Differences in the morphology of the surface of lithium after prolonged cycling demonstrates the protective nature of FGI since formation of HSAL is suppressed, however, several spots where the FGI is cracked are observed (Fig. 4b). Cracks can be induced with the dimensional changes during the stripping and deposition process (approximate change in the thickness of lithium is 10 μm per cycle). Due to cracks, the electrolyte can penetrate bellow the FGI which can induce the formation of HSAL.

**Figure 5:**
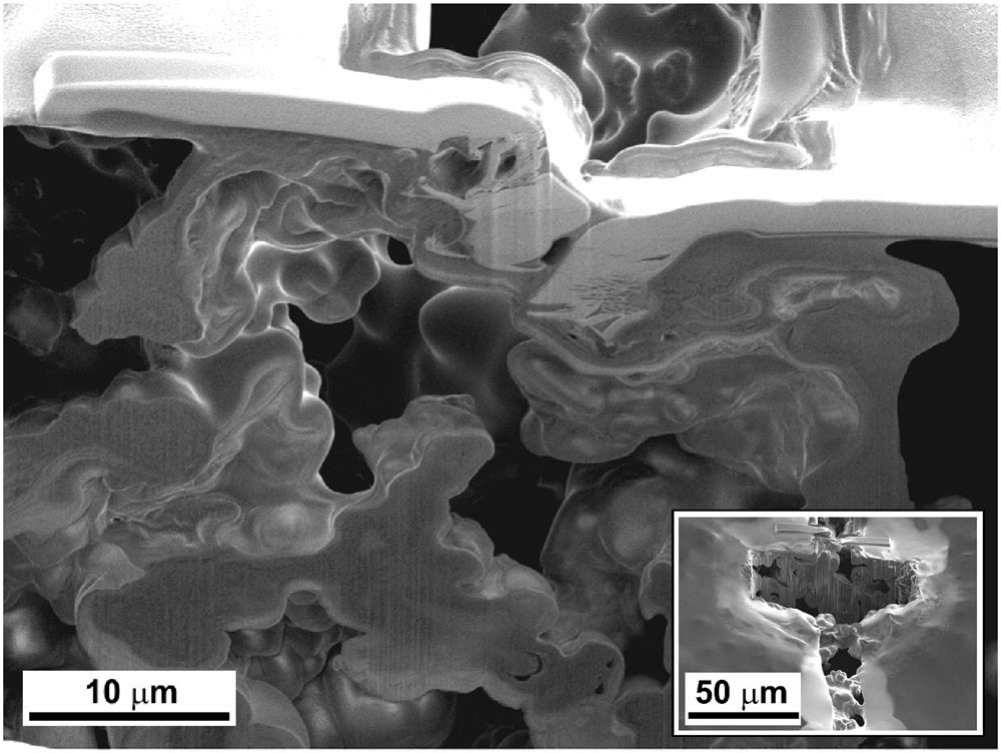
Cross-sectional view of a crack on FGI@Li. FIB-SEM image of the FGI@Li electrode after 100 cycles of stripping/deposition. Inset is a zoomed-out area of the crack where analysis by FIB-SEM was carried out.

The FIB-SEM technique was used for investigation of the morphological changes of lithium in the cracks of the FGI@Li electrode. Figure 5 shows a cross-sectional analysis of the cracked area after 100 cycles. A similar morphology as observed on the surface of non-protected lithium can also be observed in the area of crack formation, while the bulk of FGI protected lithium metal is preserved as described above and schematically shown in Fig. 2c. Due to the direct exposure of lithium to the electrolyte in the cracks, thin and unstable SEI is formed where new fresh lithium is deposited, leading to the formation of HSAL. Interestingly, no lithium deposits (dendrites or mossy lithium) were observed on the outer side of the FGI.

**Figure 6:**
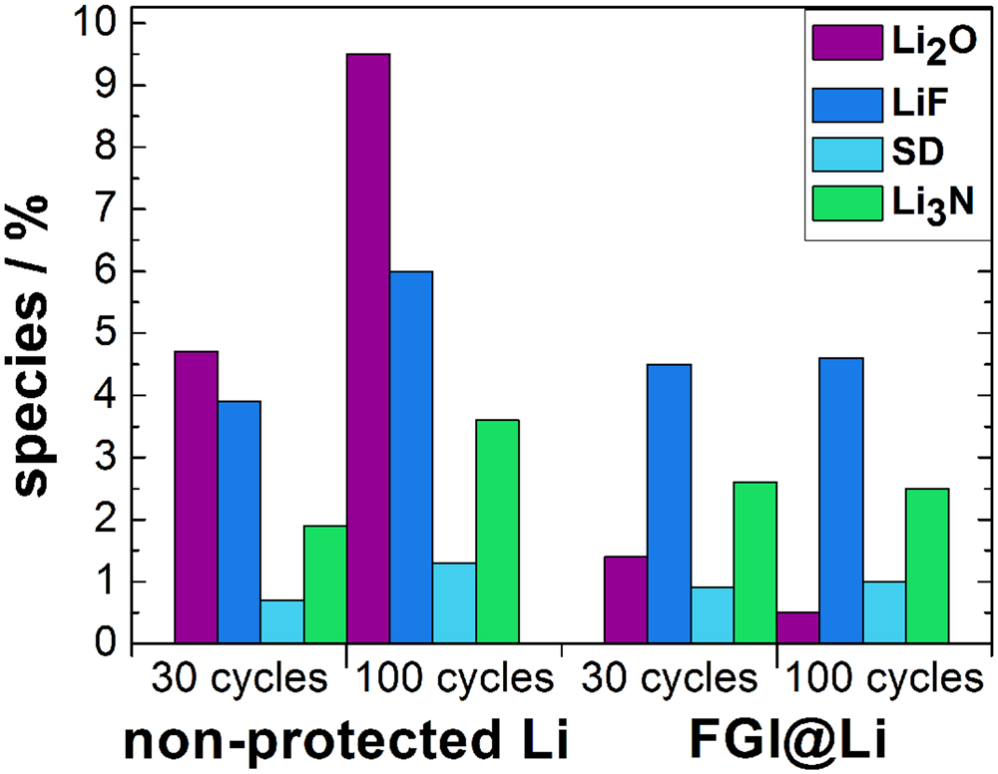
Graphic representation of XPS results. The ratio of the decomposition products as detected by XPS determined on the surface of the non-protected lithium and the FGI@Li after 30 and 100 cycles.

We applied XPS for the determination of the electrolyte decomposition products on the lithium surfaces. XPS analysis was done on the non-protected lithium and FGI@Li electrodes after 30 and 100 cycles. We confirmed the typical decomposition products that are formed in 1 M LiTFSI in TEGDME and DOL electrolyte, i.e. LiF, nitrides, and sulfur degradation products (SD) (Supplementary Figure S7). Furthermore, on the fresh non-protected lithium and FGI@Li samples, no LiF, nitrides, and SD were detected. The XPS analysis shows that the ratio of the decomposition products on the non-protected lithium surface almost doubles after 100 cycles compared to analysis performed after 30 cycles (Fig. 6). This increase of electrolyte degradation products indicates the presence of unstable SEI and decomposition of the electrolyte on the lithium surface. When FGI@Li is used, the concentration of decomposition products after 30 cycles is similar to the concentration observed on the non-protected lithium after 30 cycles. In contrast, no increase in the decomposition products is observed after 100 cycles. The stability of decomposition products was assigned to the formation of the stable SEI on the top of FGI@Li in the formation cycles as a classical process known from the graphitic materials^47^.

### Lithium metal batteries

**Figure 7:**
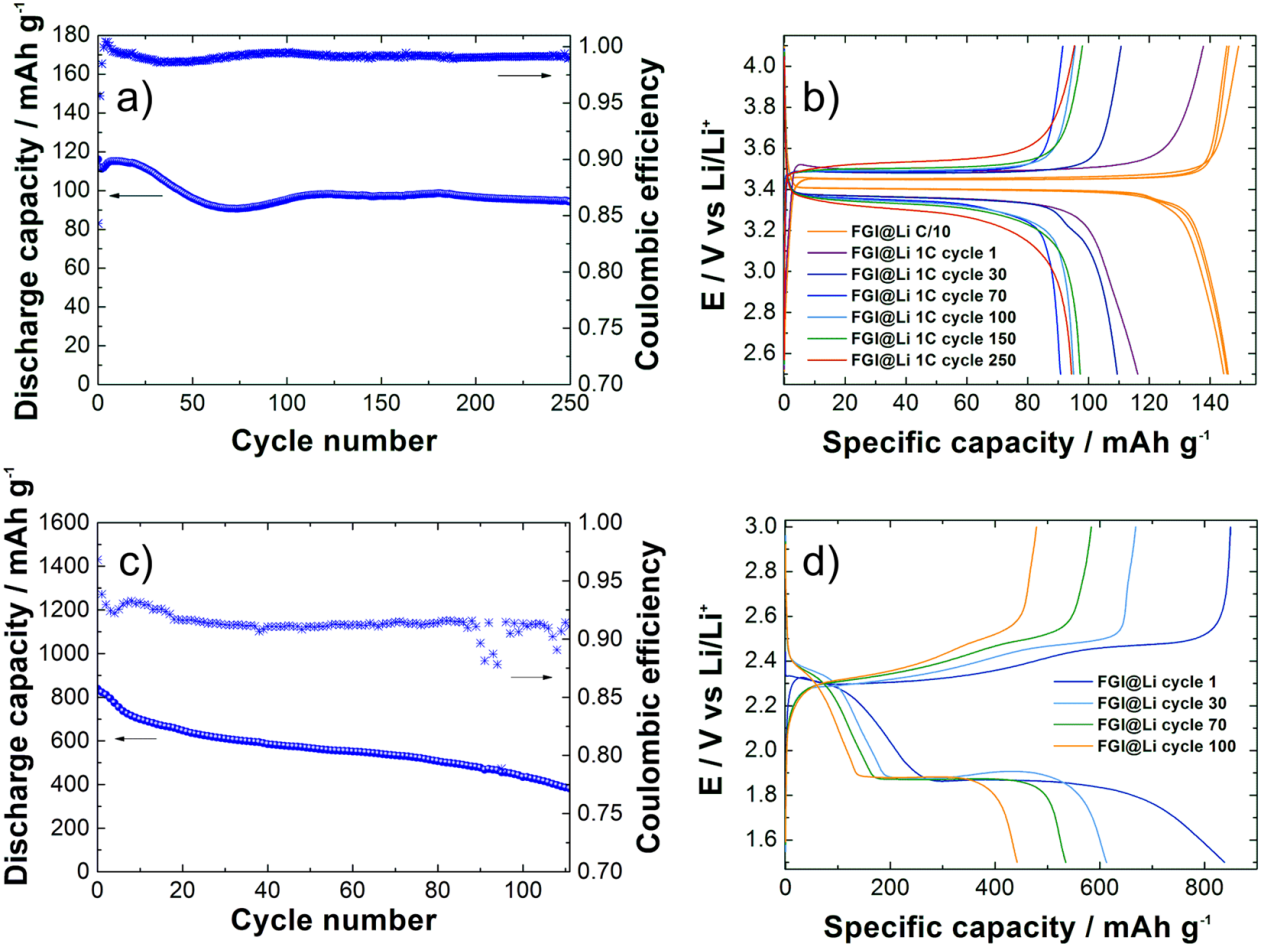
Electrochemical characterization of FGI@Li in the full-cell configuration. (**a**) Cycling performance (left y-axis) and Coulombic efficiency (right y-axis) for FGI@Li in the combination with LFP cathode, (**b**) charge/discharge curves for the LFP cathode cycled with FGI@Li, (**c**) cycling performance (left *y*-axis) and Coulombic efficiency (right *y*-axis) for FGI@Li in combination with the sulfur cathode, (**d**) charge/discharge curves for the sulfur cathode cycled with FGI@Li.

To show the applicability of FGI, FGI@Li anodes were further combined with two different types of cathode materials. LiFePO_4_ (LFP) cathodes with a loading of 4.5 mg cm^−2^ (areal capacity of 0.764 mAh cm^−2^) were tested in the carbonate-based electrolyte. Figure 7a shows charge/discharge profiles of the LFP battery combined with the FGI@Li anode. The battery was pre-cycled for three cycles at a current density of C/10, followed by continuous cycling at 1 C rate. At low C-rate we obtained almost 85% of theoretical capacity (close to 145 mAh g^−1^), while at 1 C rate the reversible capacity at the beginning was 116.2 mAh g^−1^ and it dropped to 94.4 mAh g^−1^ after 250 cycles (Fig. 7b). Small increase in the polarization can be observed during cycling which is attributed to the polarization increase on the anode side already observed during stripping and deposition tests in the symmetrical cell (Fig. 2a). The battery could be cycled for a prolonged period of time, due to the protected lithium surface, although a reasonably high loading of cathode material has been used.

Another selected configuration was a lithium sulfur (Li–S) battery, where positive electrode material is in the delithiated form and it has to be lithiated during the discharge process in the first cycle. That makes a significant difference to the lithium-metal anode, since the first process is stripping of the lithium, while using LFP, the first process was deposition of lithium to the lithium metal. Figure 7c shows the cycling performance of the Li–S battery with the cathode having a loading of 2.1 mg of sulfur per cm^2^. With an initial capacity of 800 mAh g^−1^, the areal capacity was more than 1.8 mAh cm^−2^ and we were able to prove cycling stability for more than 100 cycles. Figure 7d shows charge/discharge profiles of FGI@Li as the anode in the Li–S full-cell configuration at a current density of C/5. Again, the polarization slowly increases as already observed in the case of galvanostatic cycling of FGI@Li with LFP (Fig. 7b) and during continuous stripping and deposition electrochemical characterization (Fig. 2a).

Here we have shown that preformed FGI on the lithium surface serves as an interlayer which prevents formation of HSAL (dendrites) in two of the most commonly applied electrolyte systems (carbonate or ether-based electrolytes) and it can be used in combination with cathodes which are in the lithiated or in the delithiated form. The prolonged stripping and deposition processes, due to the high volumetric changes in the lithium electrode, generate cracks on the FGI which are responsible for the formation of HSAL below the FGI. We hypothesize that this can be improved by using polymer grafted FG which would lead to a more flexible interlayer without losing performance.

## Conclusions

We have successfully coated the lithium surface with fluorinated reduced graphene oxide (FG) by drop casting FG from the dispersion in the PC. The role of the protective interlayer on the surface of lithium (FGI@Li) was compared with non-protected lithium using stripping and deposition tests in the symmetrical cell. Prolonged electrochemical stability was obtained with FGI@Li when a high excess of electrolyte was used. The difference in the electrochemical stability was even more pronounced when comparison was performed with a reduced amount of the electrolyte, where the overpotential of the FGI@Li compared to non-protected lithium was substantially more stable and almost 50% lower. A slower increase of the overpotential can be attributed to a reduced HSAL growth and consequently slower consumption/degradation of the electrolyte for formation of the SEI layer on fresh lithium. SEM post-mortem morphology analysis on FGI@Li electrodes revealed that dendritic growth is successfully suppressed when a current density of 0.5 mA cm^−2^ is applied for 2 h. Due to high volumetric changes (approximately 10 vol.% of lithium was removed during the stripping process), formation of cracks on the FGI surface can be observed after cycling. FIB-SEM showed formation of HSAL in the vicinity of the cracks, while the bulk of the protected lithium metal is preserved. Furthermore, XPS analyses of the electrolyte degradation products revealed extraordinary stability of FGI@Li after 100 cycles compared to non-protected lithium in the lithium symmetrical cell. FGI@Li was also used in the full-cell configuration. In both tested configurations (by using LFP or sulfur cathodes), excellent electrochemical properties are demonstrated although high electrode loadings have been used. Demonstrated electrochemical cyclabiltiy of the FGI@Li electrodes in two different electrolytes, with two different cathodes, and high Young’s modulus^27^ of FGI, exhibit the potential for the utilization of fluorinated reduced graphene oxide as protective layer on the Li surface. The presented lithium protection method with some adaptation of deposition technique is fully scalable and it can be used on the industrial level. FG materials and the drop casting technique could be also used for supercapacitor and fuel-cell applications, as well as, selectivity sensors and anti-corrosion coatings.

## Experimental Section

### Materials

The propylene carbonate (PC) used to prepare the FG dispersion was obtained from Sigma Aldrich, (Lot#69896LMV, anhydrous, 99.7%) with 13.1 ppm of water content measured by Karl Fischer titration (KF, Mettler Toledo C20) stationed in an argon-filled glovebox (MBraun). Lithium foil was purchased from FMC Corporation. Custom-made electrolyte for Li-symmetrical cells and Li–S full batteries was prepared from bis(trifluoromethane)sulfonamide lithium salt (LiTFSI, Aldrich, Lot#MKBZ1840V, 99.95%), dried under reduced pressure at 150 °C for 24 h, triethylene glycol dimethyl ether (TEGDME, Lot#10199661, Alfa Aesar, 99%), and 1,3-dioxolane (DOL, Lot#34796TKV, Sigma Aldrich, anhydrous, 99.8%) both dried with a Na/K alloy under reflux. The measured water content by KF after drying TEGDME and DOL was 1.7 ppm for TEGDME and 0.2 ppm for DOL. For the Li-LiFePO_4_ full-cell batteries, a commercial electrolyte LP40 (1 M LiPF_6_ in ethylene carbonate (EC) and diethyl carbonate (DEC) in volume ratio 1:1, Merck, water content less than 20 ppm) was used.

### Synthesis

Graphene oxide (GO) and reduced graphene oxide (rGO) were prepared according to previously described methods^48,49^. The rGO was fluorinated in perfluorinated ethylene-propylene (FEP) reaction vessels as previously described^39^. To prepare the fluorinated reduced graphene oxide (FG) dispersion, FG powder (0.2 mg) was dispersed in PC (1 mL) by ultrasonication (ultrasound bath Iskra Sonis 4GT) for 4 to 6 h. The FG was dispersed homogenously as a transparent brown dispersion in the PC. The GO dispersion was prepared with the same method as the FG dispersion described above.

### Dispersions casting on the Li-foil surface

The lithium foil (110 µm) was punched in 18 mm diameter size circles and then the FG dispersion in the PC (0.2 mg mL^−1^) was applied on the lithium surface by drop-casting (50 µL cm^−2^). FG coating on the lithium surface was dried under reduced pressure (two stage oil sealed rotary vane pump, Edwards E2M1.5, 8 × 10^−3^ mbar) at 40 °C for 12 h. The as-prepared FGI@Li were punched into the electrode with a diameter of 14 mm. The GO dispersion was casted on the lithium surface with the same method as the FG dispersion described above.

### Characterization

A field-emission scanning electron microscope (FE SEM Supra 35 VP Carl Zeiss) equipped with an energy dispersive X-ray spectrometer INCA Energy 400 (Oxford Instruments) was used to obtain SEM images and EDX analysis (calibrated by Co standard) of the morphology of the FG-modified lithium electrodes. Samples were prepared in an argon-filled glovebox and transferred in a custom made vacuum transfer holder, which is opened in the SEM chamber under reduced pressure.

Cross-sectional analysis was completed using a focused-ion beam – scanning electron microscope (FIB-SEM Helios Nanolab 650i) equipped with an energy dispersive spectrometer (X-Max 50). Initially, the surface was protected with *in situ* deposited platinum to protect the surface and prevent a curtaining effect. Samples were prepared in an argon-filled glovebox and transferred into the microscope chamber under an argon atmosphere.

### XPS analyses

To prevent the moisture/air exposure of any sample, the samples were removed from their packaging within an argon-filled glovebox (concentration of O_2_ < 0.5 ppm; concentration of water < 0.5 ppm) and placed onto the sample holder without contamination. After the electrochemical stripping/deposition test, all samples were washed by DOL in baths for 1 min four times (less than 0.4 ppm of water content) to reduce the amount of salt on the surface of the samples. XPS analyses were carried out with a Kratos Axis ultra-spectrometer using focused monochromatized Al Kα radiation (h*ν* = 1486.6 eV). The spectrometer was calibrated using the Ag 3d_5/2_ photoemission peak with a full width at half-maximum (FWHM) of 0.58 eV at 368.3 eV (binding energy), and each photoemission spectrum was recorded with a constant pass energy of 20 eV. The pressure in the analysis chamber was maintained at ~5 × 10^−9^ mbar, and the analyzed area of the samples was 300 × 700 µm^2^. Short-scan spectra were measured before and after the usual long-scan experiment to check for possible degradation of the samples’ surfaces due to exposure from the X-ray beam. The binding-energy scale was calibrated with the hydrocarbon contamination using the C1s peak at 285 eV. The core peaks were analyzed using a nonlinear Shirley-type background and the peak positions and areas were obtained by using a weighted least-squares fitting of model curves (70% Gaussian, 30% Lorentzian) to the experimental data. Quantification was performed on the basis of Scofield’s relative sensitivity factors.

### Electrochemical characterization

All cells were assembled in an argon-filled glovebox and all electrochemical measurements were carried out by a Biologic VMP–300 galvanostat/potentiostat at room temperature. Coin cells (type CR2032) were assembled with a manual crimper (Hohsen Corporation) and disassembled with a coin cell disassembling tool (Hohsen Corporation) for the post-mortem analyses.

Lithium metal symmetrical cells were assembled with polypropylene (Celgard 2400) and a non-woven polyolefin separator (Freundenberg FS2225) into a coin cell (type CR2032). FGI@Li or non-protected lithium was used as both working and counter electrode and 1 M LiTFSI in a solvent mixture of TEGDME and DOL with a volume ratio of 1:1 was used as electrolyte (80 µL per cell).

Lithium symmetrical cells with a reduced amount of electrolyte were assembled with two layers of Celgard 2320 and 1 M LiTFSI in a solvent mixture of TEGDME and DOL with a volume ratio of 1:1 or LP40 as the electrolyte (10 µL per cell). Lithium metal symmetrical cells were activated in the 1^st^ cycle by stripping and deposition with a current density of 0.5 mA cm^−2^ for 4 h corresponding to an areal capacity of 2 mAh cm^−2^ and followed by stripping and deposition with a current density of 0.5 mA cm^−2^ for 2 h corresponding to an areal capacity of 1 mAh cm^−2^.

Lithium-sulfur (Li–S) batteries were assembled with polypropylene (Celgard 2400) and non-woven polyolefin (Freundenberg FS2225) as the separator. FGI@Li was used as a counter electrode and a sulfur cathode as the working electrode with a sulfur loading of approximately 2.1 mg cm^−2^ and 1 M LiTFSI in a solvent mixture TEGDME and DOL with a volume ratio of 1:1 as the electrolyte (15 µL per mg of sulfur). Sulfur cathodes were received from the Fraunhofer Institute for Silicon Technology by mixing sulfur-impregnated carbon (sulfur loading − 66 wt.%), carboxymethyl cellulose, carbon SUPER P, carbon nanotubes, and styrene butadiene rubber (SBR) in a ratio of 85:3:7:1:4 wt.% and cast on Al foil. Li–S batteries were cycled in the potential range between 1.5 and 3.0 V in a coin cell (type CR2032) with a current density of C/5 (334.4 mA g^−1^).

Lithium ion batteries were assembled with a FGI@Li negative electrode and LiFePO_4_ (LFP) cathode as the positive electrode separated by a glassy fiber separator soaked with a LP40 electrolyte. Composite cathodes were prepared by mixing active material LFP/C, carbon black, and polyvinylidene fluoride (PVdF) with a ratio of 90:5:5 wt.%^50^. The as-prepared composite mixture was dispersed in NMP and cast on Al foil. Li-ion batteries were tested in the potential range between 2.5 and 4.1 V in a pouch cell with the first three cycles at a current density of C/10 (17.0 mA g^−1^) and further cycles with a current density of 1 C (169.9 mA g^−1^). LFP loading in the composite cathode was 4.5 mg cm^−2^ of active material.

## Electronic supplementary material

Supplementary information
