# Supplementary material for: Fluorinated reduced graphene oxide as a protective layer on the metallic lithium for application in the high energy batteries
Source: Sci Rep. 2018 Apr 11;8:5819. doi: 10.1038/s41598-018-23991-2 (PMC5895819; doi:10.1038/s41598-018-23991-2)
Supplement: Supplementary file 1 — Supplementary information [file 41598_2018_23991_MOESM1_ESM.docx]

Supplementary info

**Fluorinated reduced graphene oxide as a protective layer on the metallic lithium for application in the high energy batteries**

Jernej Bobnar^1,2^, Matic Lozinšek^3^, Gregor Kapun^1^, Christian Njel^4,5^, Rémi Dedryvère^4,5^, Boštjan Genorio^2,^*, Robert Dominko^1,5,^*

^1^National Institute of Chemistry, Hajdrihova 19, SI-1001, Ljubljana, Slovenia

^2^University of Ljubljana, Faculty of Chemistry and Chemical Technology, Večna pot 113, SI-1001, Ljubljana, Slovenia

^3^Jožef Stefan Institute, Jamova cesta 39, SI-1000, Ljubljana, Slovenia

^4^CNRS / Univ. Pau & Pays Adour, Institute of Analytical Sciences and Physical Chemistry for Environment and Materials, IPREM – UMR 5254, 64000, Pau, France

^5^ALISTORE - European Research Institute, 33 rue Saint-Leu, Amiens 80039 Cedex, France

Corresponding authors: Boštjan Genorio ([bostjan.genorio@fkkt.uni-lj.si](mailto:bostjan.genorio@fkkt.uni-lj.si)),

Robert Dominko ([robert.dominko@ki.si](mailto:robert.dominko@ki.si))

FG dispersion in propylene carbonate was casted on the silicon wafer and dried at the same conditions as the lithium-modified samples described in the exprimental section (Figure S1).


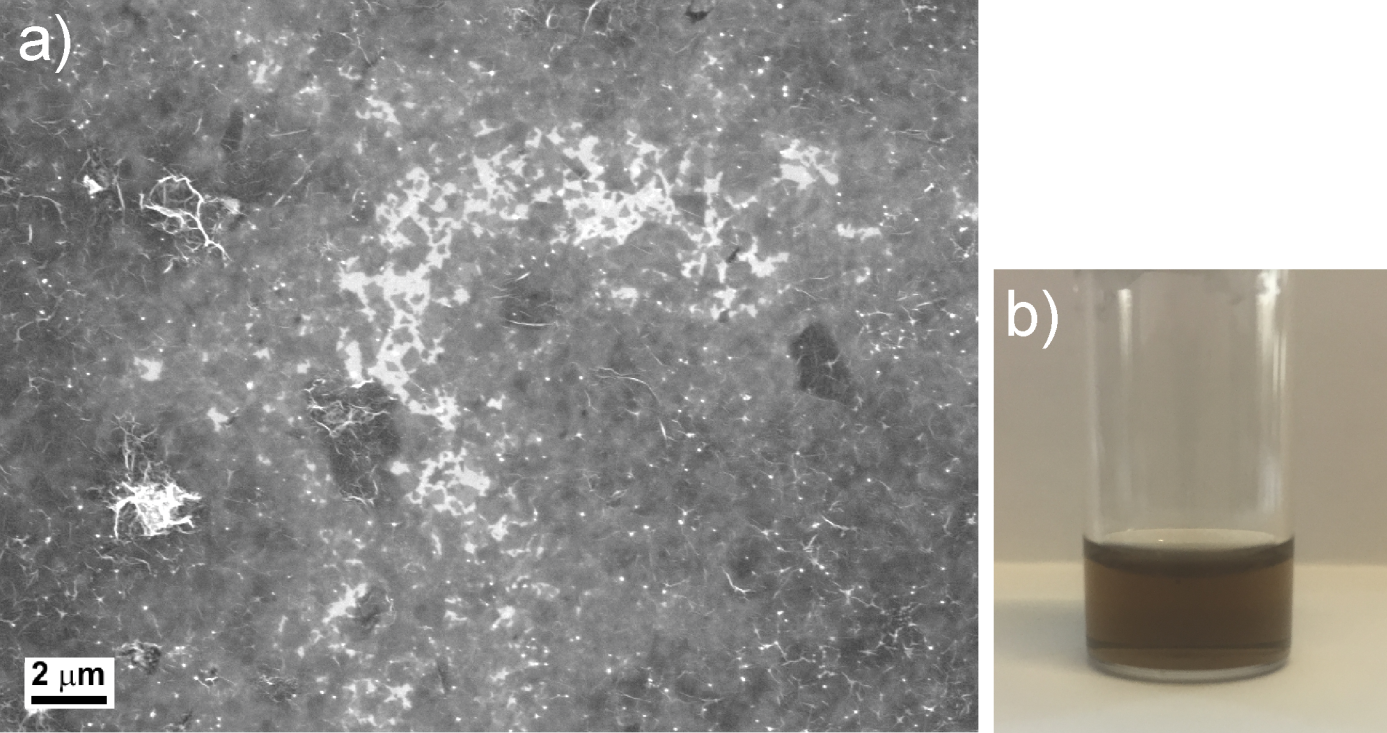


**Figure S1.** Visual characterization of FG dispersion. a) SEM image of exfoliated FG flakes on the silicon wafer and b) image of the FG dispersion in propylene carbonate.


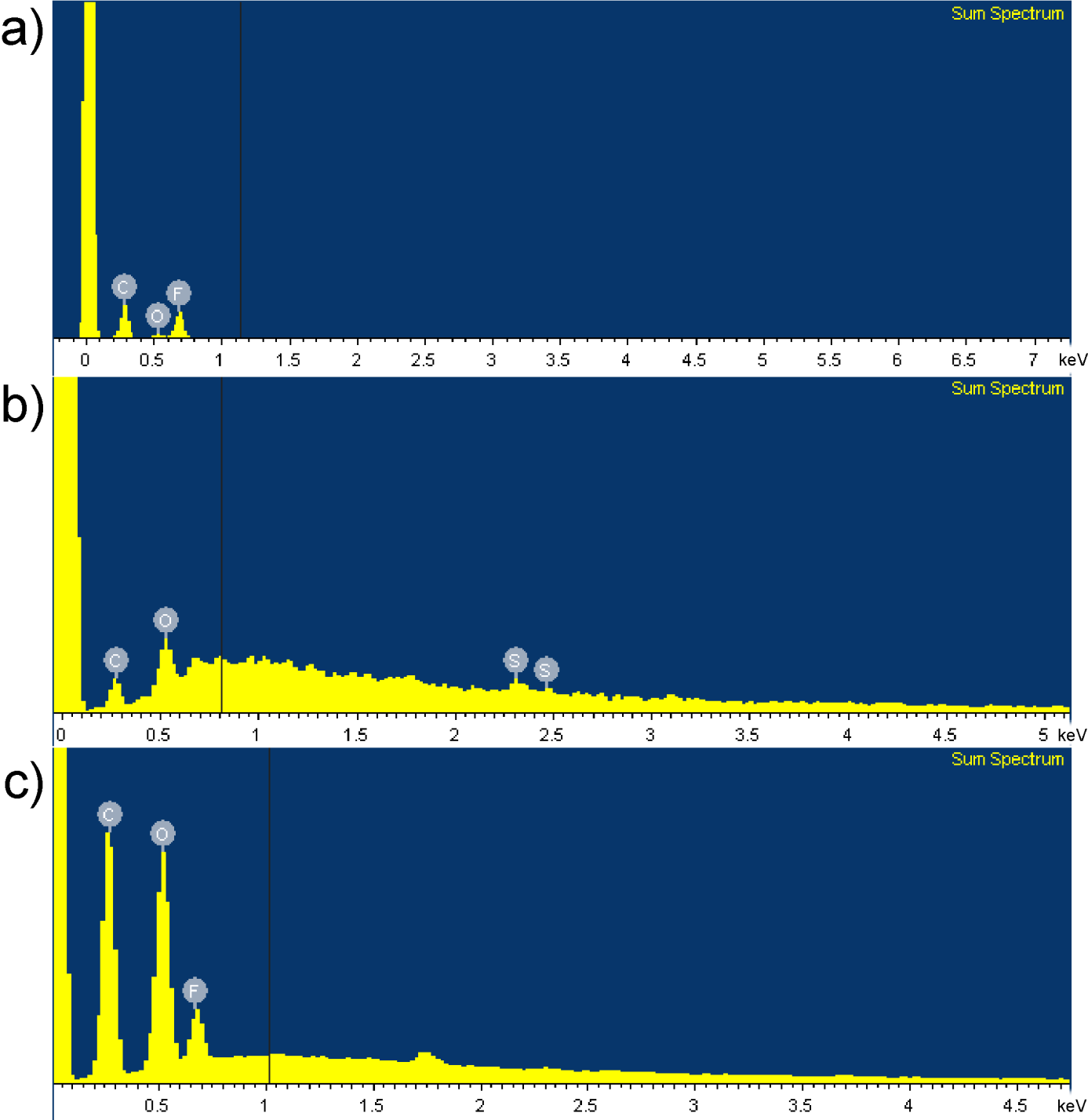


**Figure S2.** EDX analyses. a) FG flakes, b) non-protected lithium, and c) FGI@Li.

**
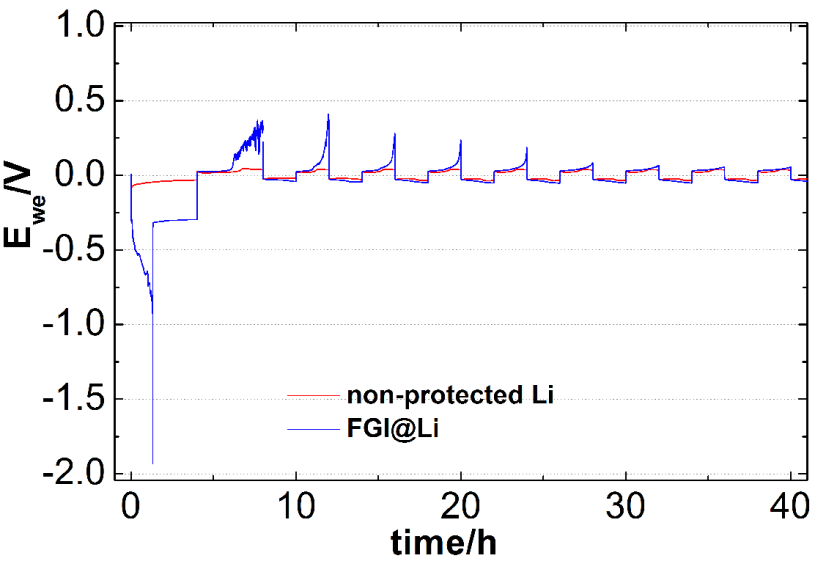
**

**Figure S3.** Voltage versus time curves in first few cycles for Li-symmetrical cells. Enlarged first few cycles of stripping and deposition test in Li-symmetrical cell for non-protected Li (red) and FGI@Li (blue) at currend density of 0.5 mA cm^–2^.


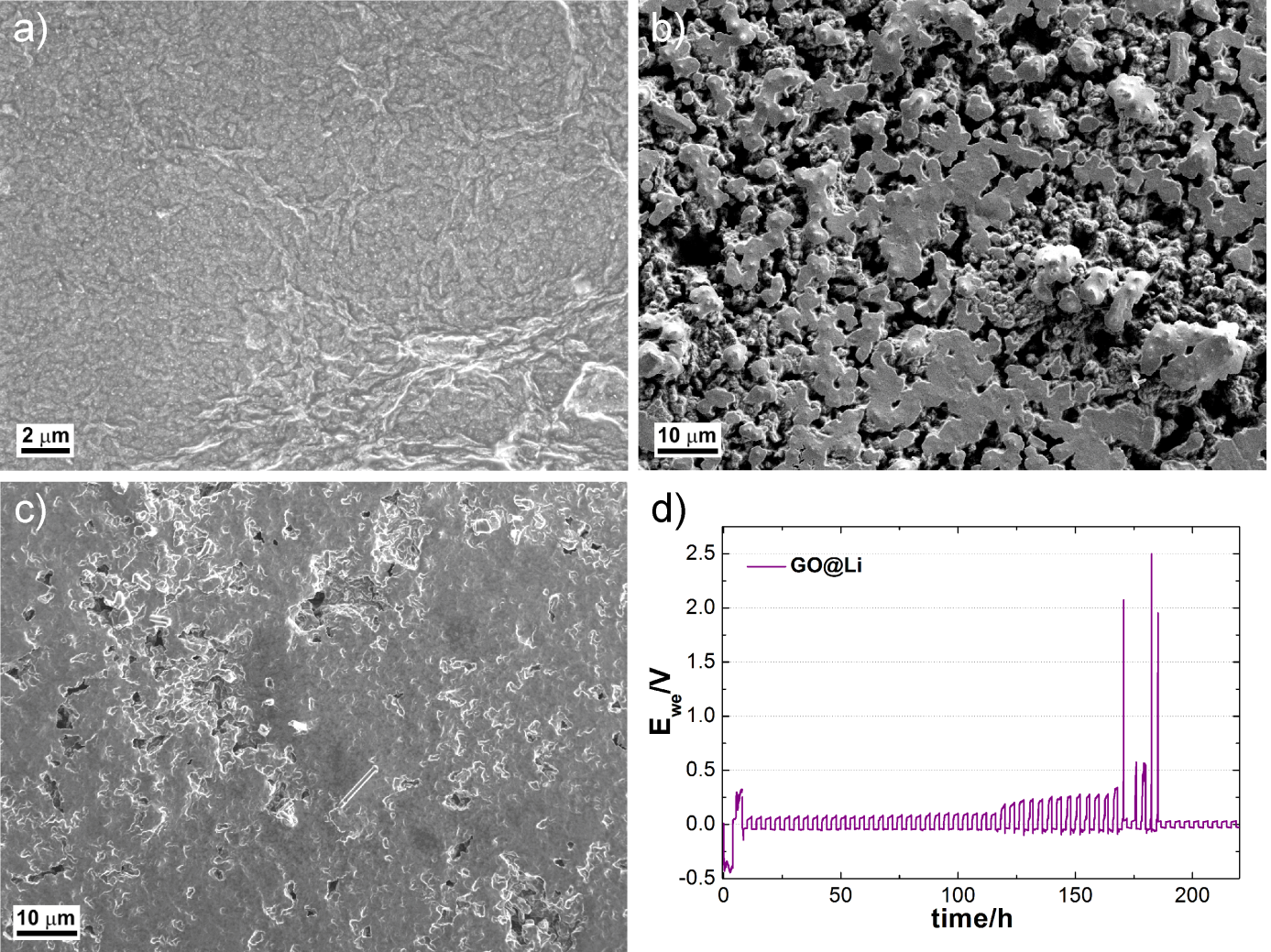


**Figure S4. Morphology of GO protected lithium and electrochemical behaviour in Li-symmetrical cell.** a) GO-coated lithium, b,c) GO-coated lithium after 30 cycles of stripping and deposition in the symmetrical cell, and d) electrochemical characterization of Li stripping and deposition of GO-modified lithium in symmetrical cell with an excess of electrolyte (80 µL per cell).


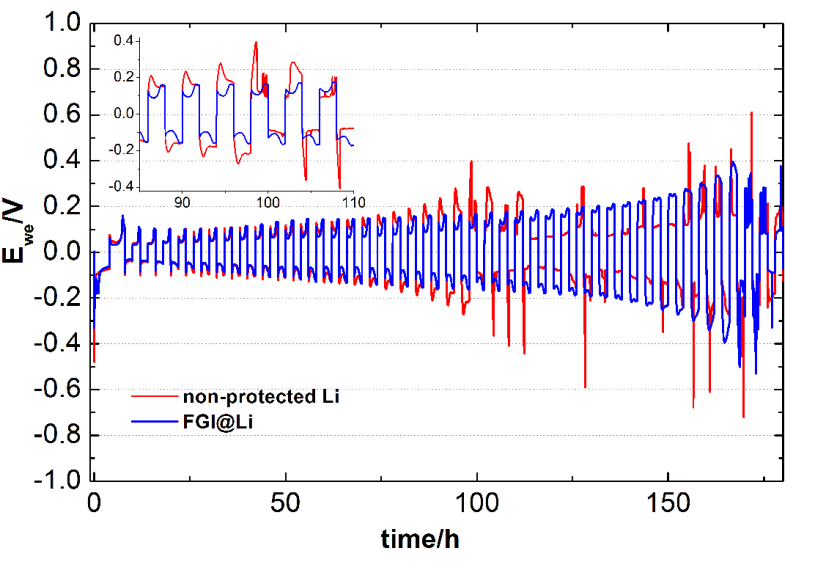


**Figure S5.** Voltage versus time curves for Li-symmetrical cells with reduced amount of electrolyte. A comparison of the electrochemical stability of the FGI@Li (blue) and non-protected Li (red) with a reduced amount of the carbonate-based electrolyte (3.3 μL cm^–2^) at a current density of 0.5 mA cm^–2^.


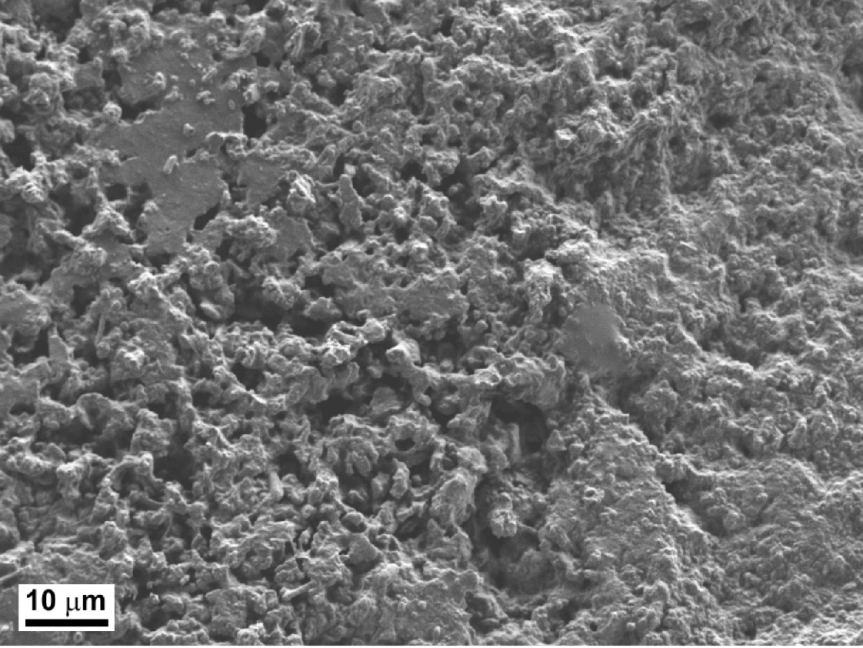


**Figure S6.** Morphology of non-protected lithium. SEM image of non-protected lithium surface after 30 stripping/deposition cycles at a current density of 0.5 mA cm^-2^.

Electrochemical salt degradation mechanism:

LiN(SO_2_CF_3_)_2_ + ne^–^ + nLi^+^→ Li_3_N + Li_2_S + Li_2_O + LiF + C_2_F_x_Li_y_ ^1–3^


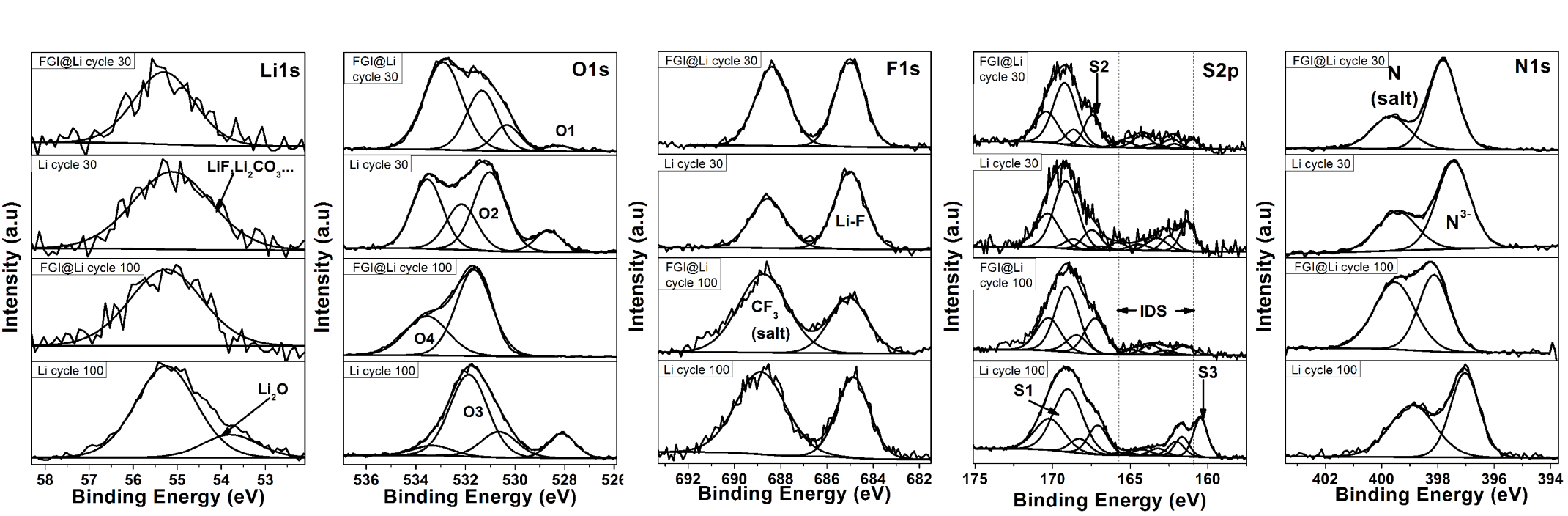


**Figure S7.** XPS spectra of non-protected lithium and FGI@Li after 30 and 100 cycles of stripping and deposition in the symmetrical cell.

Li1s spectra: one unresolved peak: lithium from various lithiated species (LiF, ROLi, Li_2_CO_3_, LiOH, ...)
O1s spectra:
- O1: oxygen from Li_2_O
- O2: oxygen from LiOH
- O3 and O4: oxygen from various oxygenated species (TEGDME and DOL degradation species, ROLi, ...)
S2p spectra:
- S1: sulfur salt (LiTFSI)
- S2: sulfur from sulfites
- S3: sulfur from Li_2_S
- IDS: intermediate degradation species

**References**

1. Xu, C., Sun, B., Gustafsson, T., Edström, K., Brandell, D. & Hahlin, M. Interface layer formation in solid polymer electrolyte lithium batteries: an XPS study. *J. Mater. Chem. A* **2,** 7256 (2014).

2. Cheng, H., Zhu, C., Lu, M. & Yang, Y. Spectroscopic and electrochemical characterization of the passive layer formed on lithium in gel polymer electrolytes containing propylene carbonate. *J. Power Sources* **173,** 531–537 (2007).

3. Vizintin, A., Lozinšek, M., Chellappan, R. K., Foix, D., Krajnc, A., Mali, G., Drazic, G., Genorio, B., Dedryvère, R. & Dominko, R. Fluorinated Reduced Graphene Oxide as an Interlayer in Li–S Batteries. *Chem. Mater.* **27,** 7070–7081 (2015).
